# Effect of Narrowband UV-B Irradiation on the Growth Performance of House Crickets

**DOI:** 10.3390/foods11213487

**Published:** 2022-11-02

**Authors:** Marios Psarianos, Anna Fricke, Shikha Ojha, Susanne Baldermann, Monika Schreiner, Oliver K. Schlüter

**Affiliations:** 1 Quality and Safety of Food and Feed, Leibniz Institute for Agricultural Engineering and Bioeconomy (ATB), Max Eyth-Allee 100, 14469 Potsdam, Germany; 2 Food4Future (F4F), C/O Leibniz Institute of Vegetable and Ornamental Crops (IGZ), Theodor-Echtermeyer-Weg 1, 14979 Grossbeeren, Germany; 3 Department Plant Quality and Food Security, Leibniz Institute of Vegetable and Ornamental Crops (IGZ), Theodor-Echtermeyer-Weg 1, 14979 Grossbeeren, Germany; 4 Faculty of Life Sciences, Food, Nutrition and Health, Professorship for Food Metabolome, University of Bayreuth, Fritz-Hornschuch-Straße 13, 95326 Kulmbach, Germany; 5 Department of Agricultural and Food Sciences, University of Bologna, Piazza Goidanich 60, 47521 Cesena, Italy

**Keywords:** resilient food systems, alternative food source, urban rearing, edible insects, LED/narrowband UV-B

## Abstract

Indoor co-cultivation systems can answer to the need for sustainable and resilient food production systems. Rearing organisms under light-emitting diodes (LEDs) irradiation provides the possibility to control and shape the emitted light spectra. UV-B-irradiation (280–315 nm) can positively affect the nutritional composition of different plants and other organisms, whereas information on edible insects is scarce. To evaluate the potential effect of the photosynthetically active radiation (PAR) and LED-emitting LEDs on the rearing and nutritional quality of edible insects, house crickets (*Acheta domesticus*) were reared from the age of 21 days under controlled LED spectra, with an additional UV-B (0.08 W/m^2^) dose of 1.15 KJm^2^ d^−1^ (illuminated over a period for 4 h per day) for 34 days. UV-B exposure showed no harm to the weight of the crickets and significantly increased their survival by ca. 10% under narrowband UV-B treatment. The nutritional composition including proteins, fat and chitin contents of the insects was not affected by the UV-B light and reached values of 60.03 ± 10.41, 22.38 ± 2.12 and 9.33 ± 1.21%, respectively, under the LED irradiation. Therefore, house crickets can grow under LED irradiation with a positive effect of narrowband UV-B application on their survival.

## 1. Introduction

It is estimated that by 2050, the world population will reach 10 billion people [1]. With many people facing hunger already, global food production needs to increase in the future [2]. However, agriculture is a major contributor to the greenhouse gas emissions [3] and water consumption [4], while its impact on soil degradation can lead to scarcity of arable land [5]. There is a growing interest in resilient food systems since food security can be disrupted and strongly affected by many unpredictable factors [6].

A possible solution lies in the further development of urban, peri-urban, or rural agricultural systems with sustainable practices [7,8]. Environmental controlled systems, allowing the co-cultivation of different organisms, including plants and animals, provide a potential response to the need for a sustainable and qualitative production system [9].

Driven by technical developments such as narrowband light emitting diodes (LEDs), indoor cultivation of plants and crops is advancing [10,11]. Artificial narrowband emitting UV/LEDs have been tested for the cultivation of several organisms, affecting the presence of secondary plant metabolites including phenolics, carotenoids and glucosinolates [12] and modulating the phenolic content of algae [13].

Diversifying crops and animal products by including alternative food sources such as insects can help build resilient food systems with increased yields and more stable national and regional value chains [14,15]. Due to their low environmental impacts [16] and high nutritional value [17], edible insects are considered a suitable alternative food sources in the future [18]. Given their comparatively easy rearing [19], high reproduction rate and short oviposition period [20], and more importantly their highly valuable nutritional profile, with a high lipid content and a protein content that can reach up to 70% [17], house crickets (*Acheta domesticus*) are attractive for introduction into future food systems. This species of edible insect is already being used as feed and food [21] and were recently accepted as a novel food under Regulation (EU) 2015/2283 [22]. House crickets offer several advantages due to their low feed conversion ratio (<2) [23,24,25], high percent that is available for food utilization with respect to conventional livestock and low water requirements [21]. Furthermore, house crickets are also reported to contain bioactive compounds, e.g., phenolic compounds [26] and vitamins [27].

Even though irradiation can play a crucial part in maintaining circadian rhythms [28] and edible insects, including house crickets, have been shown to synthesize vitamin D due to exposure to UV-B irradiation [29], the effect of artificial irradiation on their rearing circle has not been thoroughly explored yet. The present study aims to explore the possibility of introducing house crickets to an indoor co-cultivation system, by testing their response to an artificial LED-emitted illumination system, implemented in the rearing of house crickets with and without simultaneous UV-B exposure. The potential impact of this LED/narrowband UV-B irradiation regime on the nutritional composition and growth parameters of the insects was evaluated.

## 2. Materials and Methods

### 2.1. Experimental Organisms and Rearing Conditions

Adult crickets were purchased from TropicShop (Nordhorn, Germany) and reproduced at the lab-scale rearing facility at the Institute for Agricultural Engineering and Bioeconomy (ATB) so that the crickets could be raised from birth and their complete life circle could be monitored and controlled. The egg-laying substrate consisted of a mixture of sand and coconut fiber (20:1) and was kept wet at all times. After hatching, crickets were placed inside a 22 L transparent polypropylene box (39 × 28 × 28 cm) and reared for 20 days at 32 °C, 70% humidity according to Fernandez-Cassi et al. [30] inside a climatic chamber (WK-600/40 Weiss, reQutec, Borken, Germany), while exposed only to natural sunlight, transmitted through the window of the chamber. Before the LED rearing experiment and narrowband UV-B exposure, the insects were raised together until they reach the age of 20 days, in order to reach a handling size of about 0.034 ± 0.004 g/cricket and separate into different boxes. At the age of 21 days, crickets were transferred to the facilities of the Leibniz Institute of Vegetable and Ornamental Crops (IGZ) for further investigations.

### 2.2. Rearing Box Light Transparency

To test for light transparency in experimental rearing boxes, light attenuation for PAR (400–700 nm) and narrowband UV-B (280–315 nm) were determined prior experiment, using a handheld spectrometer and the corresponding software Ocean View 2.0 (Ocean Insight, Orlando, FL, USA). For this purpose, an experimental box was placed in the climatic chamber (Polyklima, Freising, Germany) exposed to intensities of PAR = 50 μmol m^−2^ s^−1^ of a 6500 K LED and narrowband UV-B = 0.04 W/m^2^ of 285 nm LED. Five different points (four corners, one center) were measured within the box, open and with closed lid, resulting in absorbance of 26% PAR and 39% narrowband UV-B, respectively. Therefore, the boxes were considered narrowband UV-B transparent, and the obtained values were used to calculate the experimental irradiation dosage.

### 2.3. LED Rearing Experiment and Narrowband UV-B Exposure

Considering the introduction of crickets to indoor co-cultivation systems, their response to LED/narrowband UV-B irradiation was explored. Ten experimental rearing boxes were placed in a climate chamber (Polyklima, Freising, Germany) in the facilities of the Leibniz Institute of Vegetable and Ornamental Crops (IGZ). The climate chamber was set to selected conditions and a photoperiod of 8h at an irradiation strength of 50 μmol m^−2^ s^−1^ of 6500 K LEDs. To avoid overpopulation that could potentially affect the rearing procedure [31], 60 crickets were placed inside each box, resulting in a total of 600 individuals. To test the potential effect of narrowband UV-B on the cricket physiology, half of the boxes (*n* = 5) were exposed to an additional narrowband UV-B dose of 1.15 KJm^−2^ d^−1^ of a 285 nm LED for a period of 34 days. As UV-B light can be used for insect pest control of plants [32] and in order not to damage the development of the insects, the light intensity was kept lower (0.08 W/m^2^) compared to the one used for plants (0.34 mW/cm^2^ = 3.4 W/m^2^) [33] or algae (470–650 µW/cm^2^ = 4.7–6.5 W/m^2^) [13]. Facing the issue of crickets hiding in the UV-B non-transparent shelter material on the 4th experimental day the narrowband UV-B irradiation time was changed. Without changing the dose, the narrowband UV-B intensity to the irradiation time was shortened from 8 to 4 h to provide higher UV-B intensities of 0.08 W/m^2^, keeping the same photoperiod of 8 h/day, at the time when fresh food was available and feeding activity was the highest. Therefore, the crickets were lured out from their hiding places and exposed as intended at least for a certain time to the experimental narrowband UV-B irradiation

During the experiment, crickets were fed three times per week with a dried commercial pellet (TropicShop, Nordhorn, Germany) and hydrogel mixed with water. To provide shelter, four pieces of egg carton were placed inside each box and changed weekly, while each box was cleaned daily to ensure hygiene.

At the experimental end at an age of 53 days, crickets were inactivated by shock freezing at −195.8 °C and stored at −80 °C until lyophilization and further processing.

### 2.4. Growth and Survival Parameters

During the experiment, two different growth parameters were estimated three times per week: (i) average weight (Equation (1)) and (ii) survival percent (SP; Equation (2)). These parameters were estimated using the following Equations of Mole and Zera [34]:

(1) $$m_{aver.}\left(g/\mathrm{cricket}\right)=\frac{m_{total}\left(g\right)}{total\;number\;of\;crickets\;}$$

(2) $$\mathrm{SP}\;\left(\%\right)=\frac{N_{i}}{N_{0}}\cdot 100\;\%$$

where ***N_i_*** is the number of crickets on day ***i*** and ***N*_0_** is the number of crickets on the first day of the measurements. The growth of the crickets was monitored from their age of 21 days, so at that point the SP was considered equal to 100%.

### 2.5. Mathematical Modeling of Cricket Growth and Survival

The values of the individual weight (g/cricket) and the SP (%) of the population were expressed as a function of the rearing time (days), with the individual weight w (g/cricket) with the time (days) being correlated with a sigmoidal equation (Equation (3)):

(3) $$w\;\left(g/\mathrm{cricket}\right)=\frac{w_{f}}{1+e^{-k\cdot \left(t-t_{0}\right)}}$$

where ***w_f_*** (g/cricket) is the weight of the crickets at the end of the rearing circle when crickets are harvested, ***t***_0_ (s), which is the sigmoid midpoint of the curve, represents the time that the first crickets reached adulthood and ***k*** (1/s) represents the growth rate.

The mathematical model used to correlate the SP (%) with the time (days) was an exponential decay equation (Equation (4)):

(4) $$\mathrm{SP}\;\left(\%\right)=a\cdot e^{-k\cdot t}+SP_{f}$$

where ***SP_f_*** (%) is the SP (%) at the end of the rearing circle, ***k*** (1/s) is the rate of reduction of the SP (%) during the rearing and a is a constant variable. Apart from Equations (3) and (4), the experimental data were expressed as functions of time using several equations, including linear, quadratic, inverse, power, exponential and logarithmic equations. Additionally, the logarithm of the values of the survival percent (%) was modeled as a function of time with a linear equation. However, none of these equations had the high value of regression coefficient and repeatability of Equations (3) and (4). The software used for constructing the models was IBM SPSS Statistics 23 (IBM Corp., Armonk, NY, USA).

### 2.6. L Composition Analysis

Freeze-dried crickets were milled into a fine powder using a Retsch GM 200 Mill (Retsch GmbH, Haan, Germany). The Ash content (*n* = 5) of the sample was estimated gravimetrically, after placing the samples at 550 °C and measuring the difference in weight. Moisture content (*n* = 5) was determined by placing the samples at 105 °C for 48 h and measuring the difference in weight. The protein content (*n* = 15) of the samples was estimated after hydrolyzing the samples with 6 N, HCl for 24 h at 98 °C and estimating the free amino nitrogen on the hydrolysates [35]. Fat content (*n* = 5) was estimated gravimetrically using the Folch method by mixing the material with a chloroform/methanol (2:1) solvent for 1 h, centrifuging and adding water to the supernatant at a volume of 0.2-times of the supernatant volume and mixing for another 30 min [36]. Chitin content (*n* = 15) was calculated by measuring glucosamine and N-acetyl-glucosamine, after hydrolysis with dilute sulfuric acid [37]. The results were expressed on a dry matter basis. For determination of the total phenolic content (TPC) (*n* = 15), 0.5 g of sample was mixed with 5 mL in an 80% methanol solution and the mixture was homogenized for 4 min. After centrifugation, the supernatant was collected, and the pellet was mixed with a 70% acetone solution. The mixture was homogenized for 4 min and after centrifuging the supernatant was collected. The two supernatants were mixed, and the liquid was removed with a rotary evaporator R-100 (Büchi, Flawil, Switzerland). The remaining extract was solubilized in 5 mL ethanol, filtered with a 0.45 µm filter and the TPC of the liquid was determined with the Folin–Ciocalteau method [38]. The TPC was expressed as mg GAE/100 g dry matter. All chemicals were of analytical grade and were purchased from Carl Roth GmbH (Karlsruhe, Germany).

### 2.7. Statistical Analysis

The individual weight (g/cricket) and SP (%) were analyzed with a Linear Mixed Model Analysis (LMM), which considered both the irradiation regime and the time (days) as fixed terms and the (irradiation*time) as a random term. The SP (%) did not follow a normal distribution and therefore was normalized with IBM SPSS Statistics 23 (IBM Corp., Armonk, NY, USA) prior to the analysis. The constant parameters of the models, as well as the values of the composition analysis were compared with an Analysis of Variance (ANOVA) with a level of significance of 0.05. Levene’s test was used to test homogeneity. The software used for all analyses was IBM SPSS Statistics 23 (IBM Corp., Armonk, NY, USA). The tables of the output of the statistical analysis are given as supplementary material.

## 3. Results and Discussion

### 3.1. Rearing of Crickets under Different Light Regimes

**Figure 1:**
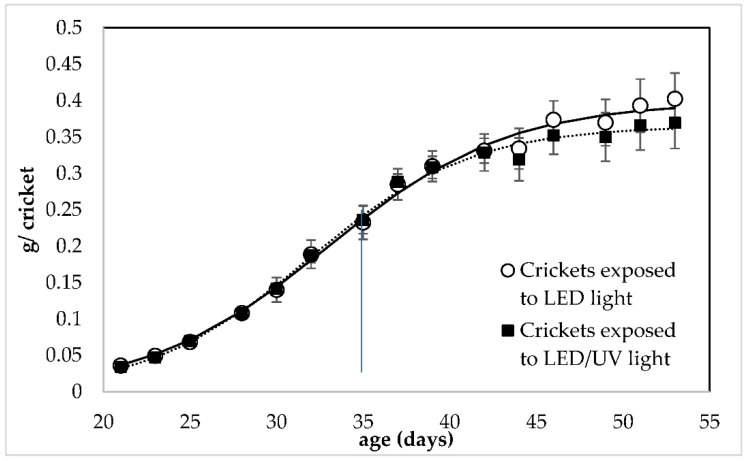
Growth of *Acheta domestica* (individual weight) reared under LED irradiation without (○) and with narrowband UV-B at 285 nm (▪). Error bars represent the standard deviation of multiple replications of the measurements (*n* = 5). Dashed lines represent the fitting of experimental data to Equation (3). The small line that is vertical to the *x*-axis indicates the time when crickets reached adulthood (35 days of age).

Both irradiation regimes led to the growth of crickets with almost equal individual weight in all replicates of all rearing systems (Figure 1). Both irradiation regimes led to the growth of crickets with almost equal individual weight in all replicates of all rearing systems. According to the Linear Mixed Model Analysis (LMM) analysis (Tables S1 and S2), the UV irradiation had no significant effect on the individual weight of the crickets (F = 3.317, df = 1, *p* = 0.076). At the end of the rearing, crickets reared under LED/narrowband UV-B irradiation regime at 285 nm had an individual weight of 0.402 ± 0.035 and 0.369 ± 0.035 g/cricket, respectively.

A significant effect of the irradiation type on the survival of the crickets was observed (F = 4.82, df = 14, *p* = 0.031, Tables S3 and S4). At the crickets’ age of 35 days, when the first adults were observed, the crickets exposed to LED irradiation showed a SP of 74.67 ± 7.83%, while the crickets exposed to the LED/narrowband UV-B irradiation showed a SP (%) of 78.63 ± 4.63%. At the end of the rearing, crickets reared under LED and LED/narrowband UVB irradiation regime at 285 nm showed a SP of 61.53 ± 4.61 and 70.51 ± 8.53%, respectively. UV-B irradiation at 285 nm did not affect (*p* > 0.05) the number of adult crickets that were harvested. In the two rearing systems with LED and LED/narrowband UV-B irradiation at 285 nm, the number of crickets that reached adulthood was 91.24 ± 4.65% and 94.30 ± 4.82%, respectively.

**Figure 2:**
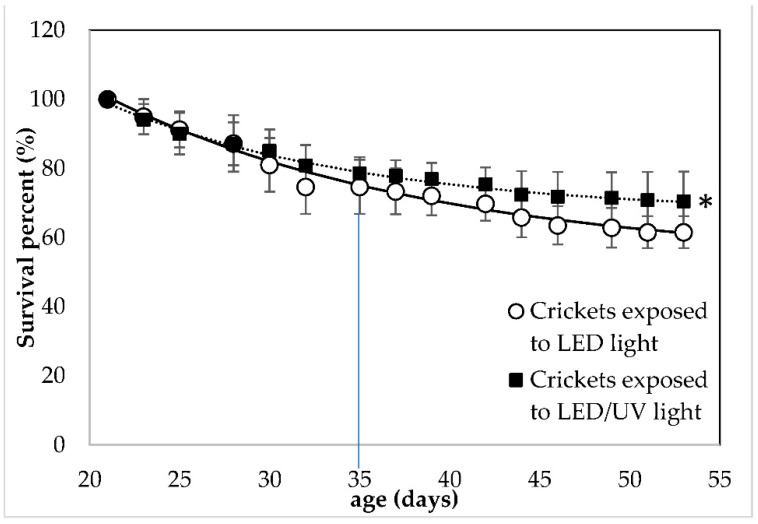
Survival percent (%) of *Acheta domestica* reared Under LED without (○) and with narrowband UV-B irradiation at 285 nm (▪). Error bars represent the standard deviation of multiple replications of the measurements (*n* = 5). Dashed lines represent the fitting of experimental data to Equation (4). The small line that is vertical to the *x*-axis indicates the time when crickets reached adulthood (35 days of age) and (*) indicates significant differences between data obtained from different treatments.

The present study investigated the response and growth performance of house crickets reared under a LED/narrowband UV-B regime that could be implemented in a co-cultivation system. Crickets were successfully reared under the LED/narrowband UV-B irradiation regime at 285 nm. The narrowband UV-B at 285 nm did not affect the weight, growth and the nutritional value of the crickets; however, it showed a positive effect on their survival (16.67% higher survival). This result indicates that the LED/narrowband UV-B irradiation at 285 nm did not cause any damage to the crickets. On the contrary, it can be implemented in their rearing system. The values of the individual weight and survival of the crickets reported by the present study (Figure 1 and Figure 2) are within the range reported by other studies focusing on the growth performance of house crickets. Specifically, it is reported that house crickets can weigh approximately 0.4 g/cricket after 50 days of rearing [39,40] and have a 55% of survival when reared at standard conditions and harvested at adulthood [41]. This indicated that crickets reared in both treatments could grow normally using the LED regime with and without the narrowband UV-B exposure.

UV light in the UVA dominating range of 300–400 nm has been known to be used for pest control due to its lethal effect on insects, such as moths [32]. Since the aims of the present study were to focus on the effect of narrowband UV-B (285 nm) on house crickets and ensure their survival, the light intensity was kept at the minimum level possible. Furthermore, crickets had been expected to hide under the egg carton during the photoperiod [20]. Despite our efforts to coordinate feeding time and UV-B exposure, active avoidance could be a further reason for no effect on the growth of the insects. These reasons could explain the low effect of UV-B irradiation on cricket physiology. 

### 3.2. Mathematical Modeling

The parameters of Equations (3) and (4) that were applied to the experimental data obtained from the two rearing systems are presented in Table 1 and Table 2, respectively. It was observed that all parameters of Equation (3) show no significant differences between the two rearing systems (*p* > 0.05). However, regarding Equation (4), it was observed that the parameter ***SP_f_***, which re the SP at the end of the rearing was significantly higher (*p* < 0.05) when Equation (4) was to the data obtained from the crickets that were exposed to the UV-B light. Both equations had a good fitting on the experimental data, since in all cases the standard errors are low and the regression coefficient (R^2^) is high (>0.980).

The data on weight and survival obtained from each replication of rearing under each irradiation regime (*n* = 5) were fitted to Equations (3) and (4), respectively. Afterwards, the average value of the model parameters obtained for each replicate of each treatment was used as initial parameters for the model that was applied to the average values of the experimental data. In all replicates of both irradiation regimes, both Equations (3) and (4) were found to have a good fitting on the experimental data, with a high regression coefficient (R^2^ ≥ 0.985 for Equation (3) for all replicates and R^2^ ≥ 0.880 for Equation (4) for all replicates). It was, therefore, considered that both Equations (3) and (4), apart from the good fitting on the experimental data, show repeatability when applied to express the individual weight (g/cricket) and survival percent (%) of the crickets as a function of their age (d), respectively.

The parameters of Equations (3) and (4) follow the trend of the experimental data. Both ***w_f_***, and ***k***, the rate of the weight increase, showed no significant differences. Furthermore, ***t***_0_ was not significantly affected by the UV-B exposure at 285 nm. This was confirmed by the experimental observation that on the 35th day of the rearing the first adult crickets were observed. Regarding Equation (4), parameter ***SP_f_*** was significantly (*p* < 0.05) higher in the model of the SP of the crickets exposed to UV-B, which confirmed the positive effect of the narrowband UV-B exposure at 285 nm. This indicated that the LED/narrowband UV-B regime at 285 nm could facilitate the inclusion of house crickets in an indoor co-cultivation system.

### 3.3. Composition Analysis

The composition of the crickets that were harvested from the two rearing systems is presented in Table 3. The UV light had no significant effect (*p* > 0.05) on the composition of the insects. Residual moisture in the samples, after freeze-drying, was approximately 5%. All insects showed a high amount of fat that was higher than 20% on a dry basis, as well as a high amount of chitin that was estimated to be approximately 10% on a dry basis, for all samples. Finally, the protein content of the crickets exposed to LED light and LED/narrowband UV-B light at 285 nm containing 60% and 65% of proteins on a dry basis, respectively, without significant differences (*p* > 0.05).

Similarly, their nutritional composition was not affected by the crickets’ weight. UV-B light has been shown to affect the total phenolic content (TPC) of other organisms, such as the microalgae *Chlamydomonas nivalis* [13]. However, this was not confirmed for the crickets. Nevertheless, the composition of the adult house crickets estimated in the present study agrees with the one reported in the literature [17].

## 4. Conclusions

The co-cultivation of different organisms can be a response to the demand for more sustainable and resilient agricultural systems. UV-B irradiation is relevant in the indoor cultivation of plants and other organisms. Crickets could be successfully reared under artificial LED irradiation system (400–700 nm). An LED/narrowband UV-B irradiation regime operating for 8 h daily, with a 4 h narrowband UV-B exposure, did not have a significant effect on the weight or the composition of the crickets. Nevertheless, narrowband UV-B exposure resulted in enhanced survival of the crickets, offering the possibility of increasing the number of crickets at the harvesting stage. The obtained experimental data on individual weight and survival were fitted into mathematical models, which confirmed experimental findings. However, further studies are needed to understand the effect of the light quality and intensity on the crickets and try to implement the same irradiation regime for several organisms, in order to further advance the design of co-cultivation systems.

## Figures and Tables

**Table 1:** Estimates of the parameters of Equation (3) (individual weight as a function of rearing time) that were applied to the average data obtained from the cricket *Acheta domestica* rearing with the two irradiation regimes.

	*w_f_* (g/Cricket)	*k* (1/s)	*t*_0_ (s)	R^2^
Crickets exposed to LED light	0.398 ± 0.007 ^a^	0.192 ± 0.011 ^a^	32.960 ± 0.342 ^a^	0.995
Crickets exposed to LED/narrowband UV-B light at 285 nm	0.365 ± 0.005 ^a^	0.217 ± 0.011 ^a^	31.854 ± 0.273 ^a^	0.996

Parameters are expressed as mean ± SD. Superscript letters indicate significant differences in the same model parameter between the two rearing systems (*n* = 5).

**Table 2:** Estimates of the parameters of Equation (4) (survival percent as a function of rearing time) that were applied to the average data obtained from the cricket *Acheta domestica* rearing with the two irradiation regimes.

	a	*k* (1/s)	*SP_f_* (%)	R^2^
Crickets exposed to LED light	150.603 ± 19.754 ^a^	0.055 ± 0.009 ^a^	53.111 ± 3.371 ^b^	0.986
Crickets exposed to LED/narrowband UV-B light at 285 nm	138.525 ± 16.189 ^a^	0.070 ± 0.007 ^a^	66.895 ± 1.203 ^a^	0.993

Parameters are expressed as mean ± SD. Superscript letters indicate significant differences in the same model parameter between the two rearing systems (*n* = 5).

**Table 3:** Composition analysis of the *Acheta domestica* that were exposed to the two irradiation regimes. Results are expressed as a percentage of dry matter.

	Crickets Exposed to LED Light	Crickets Exposed to LED/Narrowband UV-B Light at 285 nm
Dry matter (%)	95.25 ± 0.39 ^a^	95.69 ± 0.13 ^a^
Ash (%)	4.74 ± 0.08 ^a^	4.69 ± 0.25 ^a^
Fat (%)	22.38 ± 2.12 ^a^	25.08 ± 2.23 ^a^
Proteins (%)	60.03 ± 10.41 ^a^	65.59 ± 5.38 ^a^
Chitin (%)	9.33 ± 1.21 ^a^	10.19 ± 2.05 ^a^
TPC (mg GAE/100 g)	161.22 ± 10.26 ^a^	175.88 ± 37.84 ^a^

Parameters are expressed as mean ± SD. Superscript letters indicate significant differences between the two rearing systems on the content of dry matter (*n* = 5), ash (*n* = 5), fat (*n* = 5), proteins (*n* = 15), chitin (*n* = 15) and TPC (*n* = 15).

## Data Availability

All the data can be found in this study. Any inquiries or additional data can be requested from the corresponding author.

## Supplementary Materials

The following supporting information can be downloaded at: https://www.mdpi.com/article/10.3390/foods11213487/s1, Table S1: Statistical parameters of fixed effects of LMM analysis that was performed for the individual weight of the crickets as presented by IBM SPSS Statistics 23 (IBM Corp., Armonk, NY, USA); Table S2: Statistical parameters of random effects of LMM analysis that was performed for the individual weight of the crickets as presented by IBM SPSS Statistics 23 (IBM Corp., Armonk, NY, USA); Table S3: Statistical parameters of fixed effects of LMM analysis that was performed for the survival percent of the crickets as presented by IBM SPSS Statistics 23 (IBM Corp., Armonk, NY, USA); Table S4: Statistical parameters of random effects of LMM analysis that was performed for the survival percent of the crickets as presented by IBM SPSS Statistics 23 (IBM Corp., Armonk, NY, USA).
