# Supplementary material for: Effect of Narrowband UV-B Irradiation on the Growth Performance of House Crickets
Source: Foods. 2022 Nov 2;11(21):3487. doi: 10.3390/foods11213487 (PMC9658061; doi:10.3390/foods11213487)
Supplement: Supplementary file 1 [file foods-11-03487-s001.zip › foods-1988951-supplementary.pdf]

Supplementary materials

Table S1: Statistical parameters of fixed effects of LMM analysis that was performed for the individual weight of the crickets as presented by IBM SPSS Statistics 23 (IBM Corp., Armonk, N.Y., USA).

Type III Tests of Fixed Effects[a]

Source	Numerator df	Denominator df	F	Sig.
treatment	1	134,002	3,317	,076
time	14	134,002	355,055	,000

a. Dependent Variable: weight.

Table S2: Statistical parameters of random effects of LMM analysis that was performed for the individual weight of the crickets as presented by IBM SPSS Statistics 23 (IBM Corp., Armonk, N.Y., USA).

Estimates of Covariance Parameters[a]

Parameter		Estimate	Std. Error
Residual		,000464	########
treatment * time	Variance	.000000[b]	0,000000

a. Dependent Variable: weight.

b. This covariance parameter is redundant.

Table S3: Statistical parameters of fixed effects of LMM analysis that was performed for the survival percent of the crickets as presented by IBM SPSS Statistics 23 (IBM Corp., Armonk, N.Y., USA).

Type III Tests of Fixed Effects[a]

Source	Numerator df	Denominator df	F	Sig.
treatment	1	14	4,820	,031
time	14	14	27,148	,000

a. Dependent Variable: survival.

Table S4: Statistical parameters of random effects of LMM analysis that was performed for the survival percent of the crickets as presented by IBM SPSS Statistics 23 (IBM Corp., Armonk, N.Y., USA).

Estimates of Covariance Parameters[a]

Parameter		Estimate	Std. Error
Residual		,001182	,000153
treatment * time	Variance	########	,000103

a. Dependent Variable: survival.
